# Sugar Alcohol Sweetener Production by *Yarrowia lipolytica* Grown in Media Containing Glycerol

**DOI:** 10.3390/molecules28186594

**Published:** 2023-09-13

**Authors:** Piotr Juszczyk, Anita Rywińska, Julia Kosicka, Ludwika Tomaszewska-Hetman, Waldemar Rymowicz

**Affiliations:** Department of Biotechnology and Food Microbiology, Wrocław University of Environmental and Life Sciences, Chełmońskiego St. 37, 51-630 Wrocław, Poland; anita.rywinska@upwr.edu.pl (A.R.); 118950@student.upwr.edu.pl (J.K.); ludwika.tomaszewska-hetman@upwr.edu.pl (L.T.-H.); waldemar.rymowicz@upwr.edu.pl (W.R.)

**Keywords:** mannitol, *Yarrowia lipolytica*, glycerol, fed-batch culture

## Abstract

Most of the world’s annual production of mannitol is by chemical means, but, due to increasing demand for natural sweeteners, alternative production methods are being sought. The aim of the study was to screen *Yarrowia lipolytica* yeast strains and select culture conditions for the efficient and selective biosynthesis of mannitol from glycerol. From 21 strains examined in the shake-flask culture for mannitol biosynthesis from glycerol (100 g/L), three strains were selected—S2, S3, and S4—and further evaluated in batch bioreactor cultures with technical and raw glycerol (150 g/L). The best production parameters were observed for strain S3, which additionally was found to be the most resistant to NaCl concentration. Next, strain S3 was examined in batch culture with regard to the initial glycerol concentration (from 50 to 250 g/L). It was found that the substrate concentrations of 50 and 75 g/L resulted in the highest mannitol selectivity, about 70%. The fed-batch culture system proposed in this paper (performed in two variants in which glycerol was dosed in four portions of about 50 or 75 g/L) resulted in increased mannitol production, up to 78.5 g/L.

## 1. Introduction

Increased interest in foods for particular nutritional uses, e.g., reduced calorie, low calorie, and for diabetics, leads to changes in products’ composition. This is related, on the one hand, to the increased nutritional awareness of consumers—regarding the adverse effects of consuming excessive amounts of energy from sucrose—and, on the other hand, to the extended market offer of sweeteners. An unbalanced diet contributes to many diseases, including obesity, ischemic heart disease, caries, and type II diabetes, also known as non-insulin dependent diabetes [1,2].

The most effective way to reduce the consumption of sucrose is the use of artificial sweeteners—the so-called sucrose substitutes. The high intensive sweeteners used, such as aspartame, acesulfame K, cyclamen acid and its salts, neohesperidin, and thaumatin, are much sweeter than sucrose. At the same time, these compounds have different sensory characteristics than sucrose because, in addition to the sweet taste, they are accompanied by a foreign, often undesirable aftertaste in the final product (bitter or metallic) [3,4]. All sweeteners approved for use in food in EU countries have been extensively examined for potential toxicological effects in accordance with the principles of toxicological testing of food additives [5]. However, according to the literature, their potentially harmful effect on humans and animals are widely discussed [6,7,8,9,10]. For this reason, the interest of the food industry in bulk sweeteners—polyhydric alcohols (polyols) such as erythritol, sorbitol, isomalt, maltitol, lactitol, xylitol, and mannitol, which are less sweet than sucrose—has increased significantly. For over a decade, there has been a particularly strong interest in the production of erythritol [11]. However, mannitol is also noteworthy, which, thanks to its properties, is widely used in the industry, mainly food and pharmaceutical, but also in medicine [12,13,14]. It gives a sweet taste, and at the same time shapes the consistency and texture of products. Mannitol has half the sweetness and a reduced calorific value (1.6 kcal/g) compared to sucrose (4 kcal/g) [15]. It is used in low-calorie sweeteners, in food for diabetics, because its insulin and glycemic index is “0”. It reduces the tendency of sugars to crystallize, which extends the shelf life of food products. Crystalline mannitol has a very low hygroscopicity, which is why it is used in products that require stability at high humidity [12].

According to the Global “Mannitol Market 2020–2025” Research Report, over the next five years, the mannitol market will register a 4.0% compound annual growth rate in terms of revenue, and the global market size will reach $501.1 million by 2025, from $429 million in 2019 [16]. On average, 150,000 tons of mannitol are used annually in the world, which is 11% of the total polyols used [14].

Initially, on an industrial scale, mannitol was obtained from the bark of manna ash (*Fraxinus ornus*) [17,18]. Enzymatically, mannitol can be synthesized from fructose with the catalysis of mannitol dehydrogenase (MDH) using NAD(P)H as a reducing cofactor [14,18]. However, this process has some limitations related to the cost of cofactors, enzyme purification, their stability, or inhibition of enzyme activity by a high concentration of substrate in the mixture [19]. Currently, mannitol is mainly produced by chemical hydrogenation of a mixture of glucose and fructose at high pressure (70–140 atm) and high temperature (120–160 °C) using Raney nickel as a catalyst and hydrogen gas [20]. The development of an alternative, simpler, and more economical method of mannitol production would facilitate access to this substance, primarily due to lower production costs, and thus a lower price of the final product. The results described in the literature indicate the possibility of using microorganisms to produce mannitol, which in the cell can be a reserve material or an osmoregulatory factor synthesized in response to abiotic stress [12]. Particular interest is paid to application of lactic acid bacteria (LAB) that are representatives of homo- and heterofermentative genera such as *Streptococcus*, *Lactobacillus*, *Lactobacillus*, and *Leuconostoc* [14,21,22], while among fungal genera, *Pircularia*, *Aspergillus*, *Eurotium*, and *Fennellia* have been studied [15,22]. However, the use of these microorganisms generates a number of inconveniences. *Lactobacilli* are auxotrophs that are not very resistant to changing environmental conditions and are small, while fungi form hyphae and are characterized by a long generation time. Studies also indicate the possibility of producing mannitol by the use of yeast [12,14,15,22,23]. These microorganisms can produce mannitol using glucose and fructose, but also less traditional substrates such as five-carbon sugars (e.g., *Candida*, *Brettanomyces*, *Decceromyces*, *Kluyveromyces*, *Saccharomyces*, *Schwanniomyces*, and *Trichosporon*), glycerol (*Torulopsis versatilis* and *Candida azyma*), mannose, galactose, maltose, or xylitol [22,24].

In studies conducted on the biosynthesis of citric acid by *Yarrowia lipolytica* yeast, by using technical glycerol as well as raw glycerol from biodiesel production as substrates, undesirable by-products were determined, including erythritol, mannitol, and arabitol [25]. It is known that in the cell polyols may act as osmoprotectants. Therefore, many studies have focused on application of various osmotic agents, e.g., NaCl, polyethylene glycol, or high concentration of substrate as factors that induce the production of polyols. High osmotic pressure was successfully used to increase the production of erythritol; however, mannitol production was inhibited under such conditions [26,27,28,29]. Hence, increasing the production of mannitol requires new methods and strategies, e.g., media optimization and cultivation systems or application of genetically engineered strains [11,28,29,30,31]. In literature reports, the potential of wild-type strains of *Y. lipolytica* to produce high amounts of mannitol, which accounted for up to 40% of the products, was observed [29]. Therefore, in the presented study, we aimed at screening *Y. lipolytica* yeast strains for effective mannitol production in a glycerol-containing medium by developing a substrate dosing strategy in fed-batch fermentation.

## 2. Results and Discussion

The strains of *Yarrowia lipolytica* used in this work were previously examined for the production of biomass from raw glycerol [32]. The great potential of these strains for the utilization of glycerol has already been demonstrated; therefore, in this study, they were used to produce value-added products such as polyols, which can be used as a substitute for sucrose. Growing consumer awareness, trends for a healthy lifestyle, and the search for alternative methods in medicine and cosmetology have raised interest in alternative sweeteners, among which polyols, including mannitol and erythritol, are of particular importance [33].

### 2.1. Y. lipolytica Strain Selection in Flask Cultures

**Figure 1:**
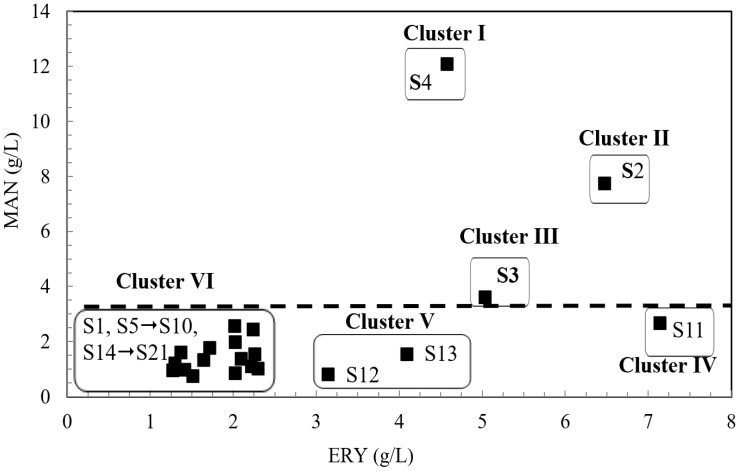
Cluster analysis of mannitol (MAN) and erythritol (ERY) concentration obtained in 7-day shake-flask experiment using 21 strains of *Y. lipolytica* (S1→S21). Culture condition: screening medium with 100 g/L of technical glycerol.

In the first stage of the study, shaken cultures were carried out, in which the capacity of 21 strains (S1→S21) of *Y. lipolytica* yeast for biosynthesis of mannitol from technical glycerol was assessed (Figure 1). It was found that all tested yeast strains were able to grow in the screening medium containing 100 g/L of technical glycerol, at pH = 3.0. Significant differences in the production of mannitol and erythritol were noted, depending on the strain used. After 7 days of cultivation, the final concentration of mannitol ranged from 0.8 g/L (strain S16) to 12.1 g/L (strain S4). The concentration of erythritol in the medium ranged from 1.3 g/L to 7.1 g/L for strain S17 and S11, respectively (Figure 1).

For comparison, in the study conducted by Vastaroucha et al. [34], the maximum concentrations of mannitol reached 19.64 and 16.11 g/L and were obtained after 48 and 96 h of cultures conducted at pH 3.0 and 4.0, for *Y. lipolytica* FMCC Y-74 and FMCC Y-75, respectively. On the other hand, in studies involving the CICC 167 strain of *Y. lipolytica* in a process lasting 120 h in a medium with an initial concentration of glycerol of 150 g/L, significantly higher concentrations of mannitol (36.8 g/L) and erythritol (41.3 g/L) were obtained at complete depletion of glycerol [28].

The results obtained in this study were analyzed using a statistical technique known as cluster analysis. Its purpose is to divide data into clusters (groups) in such a way that each of them contains objects that are most closely related to each other (similar to each other), and at the same time, elements belonging to different clusters should be as different as possible. To estimate the distance between the clusters, the Ward method, which uses the concept of analysis of variance, was used. Six clusters were obtained, in which the data were characterized by similar properties (Figure 1). The performed analysis of variance (at *p* ≤ 0.05) and the results of the Duncan test showed statistically significant differences between the strains classified into individual clusters. However, within individual clusters, no statistically significant differences in the production of mannitol and erythritol were found between individual strains (at *p* ≤ 0.05).

In group I, the S4 strain with the highest production of mannitol and erythritol was extracted, amounting to 12.1 and 4.6 g/L, respectively. A high concentration of erythritol was observed for strains S11 (group IV) and S2 (group II); however, it is worth noting that strain S2 produced mannitol in the amount of 7.8 g/L. Based on the statistical analysis of the obtained results, strains representing three clusters were selected for further research: Cluster I—S4, II—S2, and III—S3.

### 2.2. Mannitol Production in Batch Cultures

**Figure 2:**
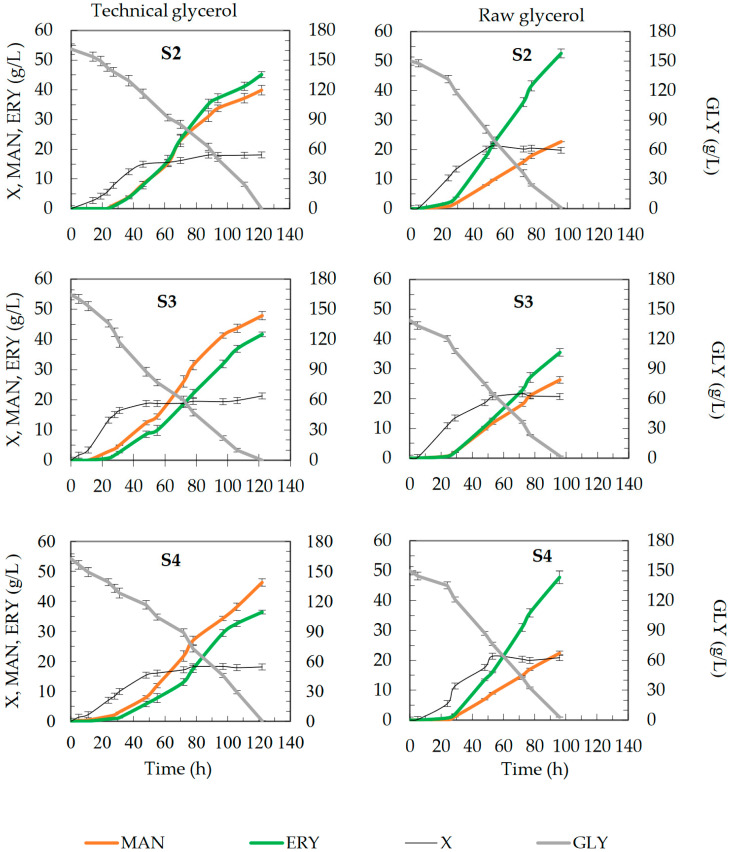
Time course of glycerol consumption (**GLY**) and biomass (**X**), mannitol (**MAN**), and erythritol (**ERY**) production by S2, S3, and S4 strains of *Y. lipolytica* yeast during batch fermentation in bioreactor cultures in production medium containing 150 g/L of technical or raw glycerol.

Due to the possibility of controlling basic culture parameters, such as temperature, pH, or aeration of the medium, the evaluation of the kinetics of the mannitol production process by strains S2, S3, and S4 was carried out in bioreactor cultures in a medium containing 150 g/L of technical or raw glycerol. The course of the mannitol biosynthesis process is presented in Figure 2, and the final results are summarized in Table 1.

The cultures were assessed until complete substrate depletion. When technical substrate was used, glycerol was depleted within approximately 122 h, and the biomass concentration in the stationary phase ranged from 18.1 to 21.3 g/L, depending on the strain (Figure 2). The final concentration of mannitol was similar for strains S3 and S4, about 46 g/L, while in the case of strain S2, it was lower, 39.9 g/L. Erythritol production ranged from 36.5 to 45.2 g/L. The use of raw glycerol resulted in a shortening of the process by about 24 h (Figure 2), with almost complete depletion of the substrate (residual glycerol concentration ranged from 0.9 to 3.0 g/L). In these cultures, there were no significant differences in the amount of biomass obtained between the strains, which ranged from 19.8 to 20.9 g/L. However, for individual strains, the biomass concentration was slightly higher than in the medium with technical glycerol. The use of raw glycerol significantly affected the total polyols and the ratio of mannitol to erythritol (Table 1). In the medium with raw glycerol, mainly the production of mannitol was significantly reduced, which resulted in a lower level of total polyols (by as much as 27.8 g/L for the S3 strain). Depending on the strain, from 22.4 to 26.3 g/L of mannitol and from 35.5 to 52.6 g/L of erythritol were obtained.

Contaminations present in raw glycerol might be responsible for the differences in the concentration of biomass and polyols, which were previously discussed [29,32]. The chemical composition of raw glycerol depends mainly on the type of catalyst used for the production of biodiesel, the efficiency of the transesterification process, the efficiency of recovery of substrates (methanol/ethanol and catalyst), and the finished product (biodiesel), as well as other natural organic and inorganic impurities present in the raw material [28,35,36]. In most cases, chemical catalysts are used, mainly NaOH or KOH; hence, the content of sodium and potassium salts can be as high as 12% [37]. The presence of salt in the raw glycerol increases the osmotic pressure, and the high osmotic pressure of the culture medium is known to be a key factor in the production of polyols by microorganisms.

Polyhydric alcohols such as glycerol, erythritol, mannitol, or arabitol are the most frequently formed osmoprotectants in the cells of some microorganisms, including the yeast *Y. lipolytica* [11,38]. However, when exposed to high osmotic pressure, *Y. lipolytica* preferred to produce erythritol rather than mannitol in order to balance the osmotic pressure between inside and outside of the plasma membrane more effectively [28].

This is in accordance with the results obtained in this study, where higher concentrations of erythritol and lower concentrations of mannitol were observed in cultures with raw glycerol that contained NaCl (Table 1). The mechanism of mannitol and arabitol production accompanying erythritol biosynthesis is still not well understood [28]. However, it is known that these compounds have a protective and stabilizing effect on enzymes, enabling them to function properly in these unfavorable conditions [39].

To summarize this stage of the research, it is worth pointing out that strain S3 in the medium with technical glycerol produced the largest amount of polyols, while in the presence of raw glycerol, it definitely produced the lowest (Table 1), which indicated different sensitivities of these yeasts to changes in osmotic pressure and suggested the need to examine the strains in a model medium containing technical glycerol and various concentrations of NaCl.

### 2.3. Effect of Osmotic Pressure on Mannitol Production

#### 2.3.1. Impact of NaCl Concentration

**Figure 3:**
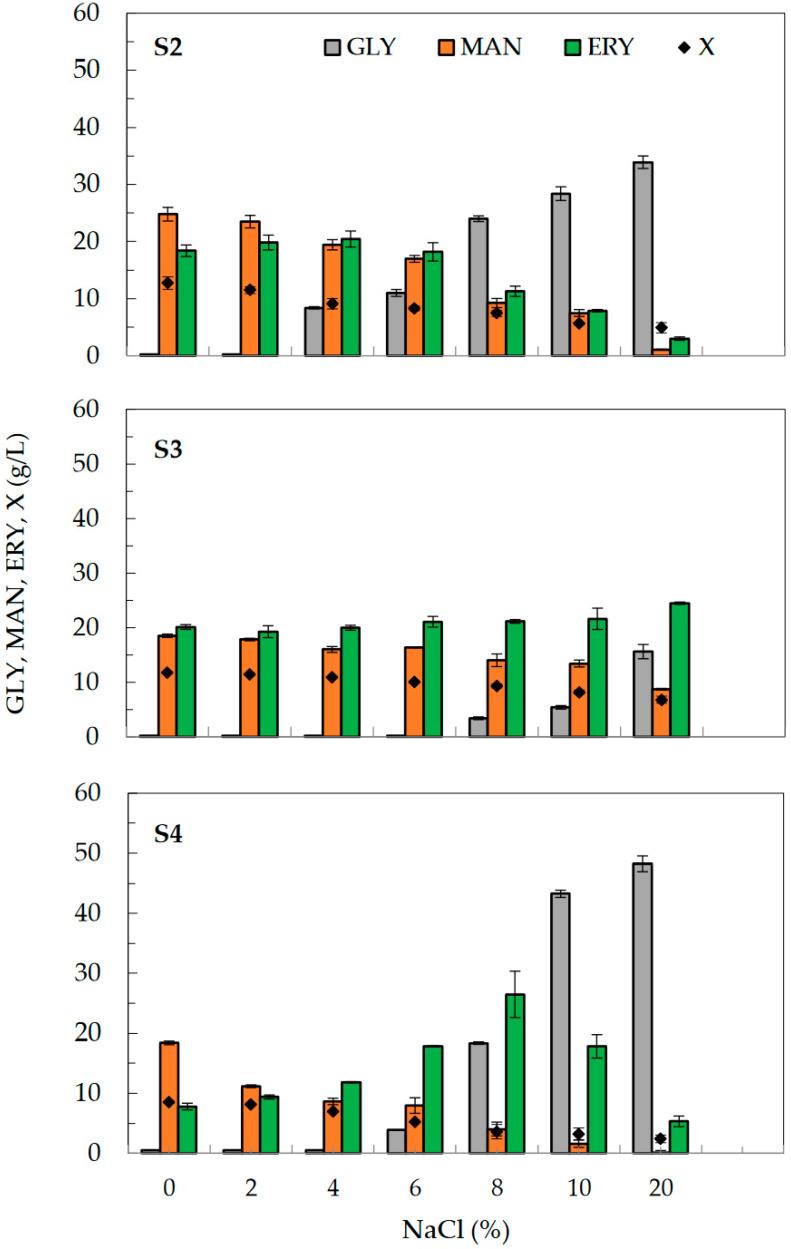
Effect of NaCl concentration (0–20%) on mannitol—MAN (**■**), erythritol—ERY (**■**), and biomass, X (♦) production by S2, S3, and S4 strains of *Y. lipolytica* yeast in 7-day shake-flask cultivation. Culture condition: screening medium with 150 g/L of technical glycerol, GLY—(■). Data are mean values of three samples, and error bars represent standard deviation of data.

In this part of the study, the effect of NaCl on the production of mannitol from technical glycerol by strains S2, S3, and S4 was examined in a 7-day shake-flask culture experiment (Figure 3). The ability of yeast to produce mannitol was evaluated in a screening medium containing 150 g/L of technical glycerol in the presence of NaCl (2 → 20%). Yeast cultures performed in medium without the presence of salt were used as the control.

The experiment results showed different tolerance of the examined strains to the presence of salt in the culture medium. The smallest variation in biomass concentration, from 6.8 to 11.8 g/L, was presented by the S3 strain, while in the case of the S2 strain, the biomass level ranged from 4.9 to 12.73 g/L. It is worth noting that strain S3 of *Y. lipolytica* investigated in this work appeared to be more halotolerant when compared to the strains of the same species isolated from blue cheeses, which were able to grow at 8.0–10% NaCl [40,41]. Ability to adapt to high salinity (25% salinity, 12% NaCl) was also observed for *Y. lipolytica* [42].

In this study, it was noted that the presence of salt in the medium affected the utilization of glycerol by yeast. In the case of the S2 strain, the presence of residual substrate was observed when NaCl concentration reached 4% or higher, whereas for the S3 strain, it was observed from 8% NaCl. The highest concentration of residual glycerol was noted in the culture of the S4 strain in the presence of 20% NaCl.

The highest mannitol amounts, from 18.4 g/L (S4) to 24.8 g/L (S2), were observed in control cultures performed without addition of salt. For strains S2 and S4, mannitol production decreased significantly with increasing salt concentration in the medium, and reached only 0.5 g/L in the case of strain S4 cultured in a medium with 20% NaCl. A similar inhibitory effect of presence of NaCl on mannitol production was described in earlier reports concerning polyol production by *Y. lipolytica* from glycerol [28,29,43]. In this study, especially noteworthy was the S3 strain, which showed the lowest sensitivity to the presence of salts in the medium. In the case of this strain, the maximum examined concentration of NaCl (20%) resulted in only a 50% reduction in mannitol production. Therefore, this strain was selected for the next stage of research, in which the effect of osmotic pressure caused by different initial glycerol concentrations was evaluated in bioreactor cultures.

#### 2.3.2. Effect of Initial Glycerol Concentration

Bioreactor batch cultures were performed in the medium with the initial concentration of glycerol from 50 to 250 g/L, which corresponded to the water activity ranging from 1.03 to 3.53 Osm/kg (Table 2). In the culture with 250 g/L of glycerol, no yeast growth was observed. In other cultures, the biomass level was in the range of 20.3–28.1 g/L, and the processes were carried out until the complete depletion of the substrate, which lasted from 45 to 100 h. The amount of total polyols produced increased simultaneously with increased substrate concentration up to 142.7 g/L in the culture containing 200 g/L of glycerol. However, the highest production efficiency (Y_ΣPol/S_ of about 0.7 g/g) was noted in media containing 150–200 g/L of the substrate (Table 2). The effect of initial glycerol concentration (50–300 g/L) on polyol production was studied in flask cultures by Yang et al. [28]. It is worth noting that the strain *Y. lipolytica* CICC 1675 examined in their study was able to grow even when as high as 300 g/L of glycerol was utilized in the medium. The authors also observed an increase in polyol production when glycerol concentration was increased up to 200 g/L; however, the total polyol concentration was 106.1 g/L, so it was lower than the level observed by us. In this study, the highest concentration of mannitol, 54.7 g/L, was obtained in the process in which the initial concentration of glycerol was 175 g/L (Table 2). It should be emphasized that, when compared with literature reports, these are the highest amounts of mannitol produced by *Y. lipolytica* yeast in batch culture so far. A slightly lower concentration of this polyol, 50.4 g/L, was reported by Yang et al. [28] for a batch culture process carried out in flasks in the medium with initial glycerol concentration of 200 g/L. In other reports concerning the production of mannitol in bioreactor batch cultures using *Yarrowia* clade yeast, the polyol concentration ranged from 0.6 to 43.0 g/L [26,29,33,44].

However, it should be noted that mannitol was not the main product of these processes, but rather a side product during the biosynthesis of erythritol or citric acid from glycerol. The possibility of mannitol production was also studied using other yeast species and different substrates, e.g., n-paraffin by *Candida zeylanoides*, glucose by *Torulopsis versatilis*, and fructose by *Candida magnoliae*, which resulted in mannitol production of 52–67 g/L [22,23]. According to the literature, the highest concentration of mannitol (223 g/L) was obtained with the use of *C. magnoliae* HH-04 in a batch culture conducted in fructose medium supplemented with Ca^2+^ and Cu^2+^ ions [45].

Summarizing this stage of the research, it should be emphasized that, regardless of the osmotic pressure produced by different substrate concentrations, mannitol predominated in the total sum of products. At the lowest concentrations of glycerol, 50 and 75 g/L, the highest selectivity of mannitol was noted and reached 80% and 68%, respectively (Table 2). Therefore, application of these concentrations of glycerol was recommended for the next step of research.

### 2.4. Polyol Production in Fed-Batch Culture

**Figure 4:**
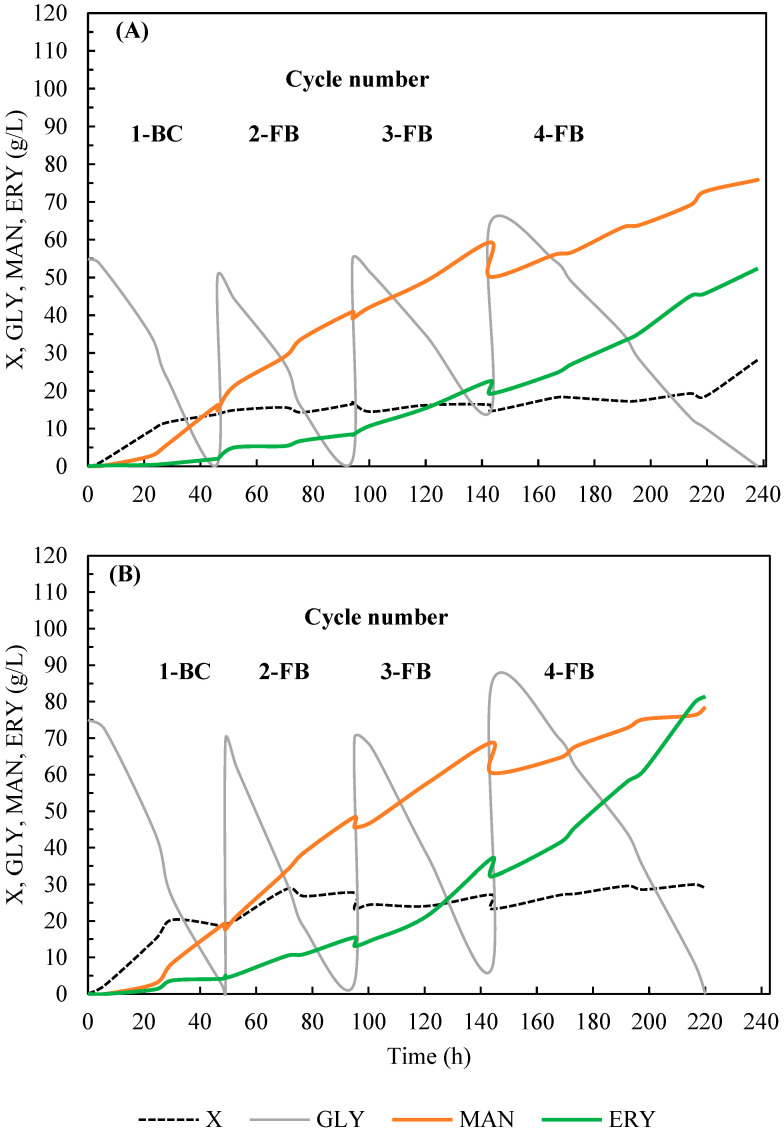
Mannitol (**MAN**), erythritol (**ERY**), and biomass (**X**) production and glycerol consumption (**GLY**) by *Y. lipolytica* S3 during fed-batch fermentation in bioreactor cultures in production medium with technical glycerol. Culture medium was fed with three portions of glycerol in order to obtain final glycerol concentrations of 200 (**A**) and 300 (**B**) g/L.

Two fed-batch cultures with different feeding strategies were performed (50 + 3 × 50 or 75 + 3 × 75 g/L of the substrate) (Figure 4). Each process was started as a batch culture with an initial glycerol concentration of 50 or 75 g/L, and next, after substrate depletion, the culture was fed with three equal doses of 50 or 75 g/L of the substrate, resulting in the total glycerol concentration of 200 g/L (Figure 4A) and 300 g/L (Figure 4B), respectively. The total time of the process, necessary for complete substrate utilization, was comparable for both variants of the feeding strategy (about 220 h).

**Figure 5:**
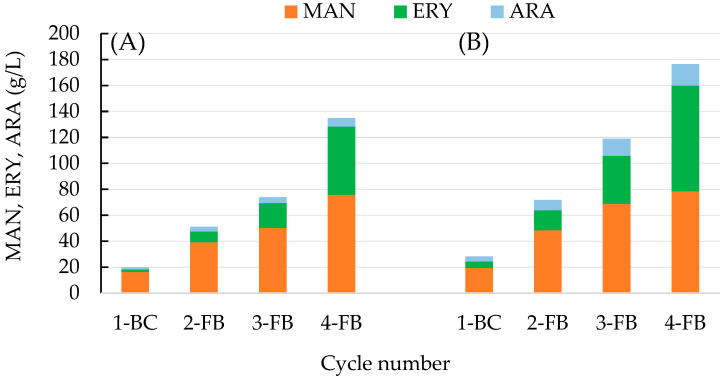
Mannitol—MAN (■), erythritol—ERY (■), and arabitol—ARA (■) production during fed-batch fermentation in bioreactor cultures of *Y. lipolytica* S3 in production medium with technical glycerol, with the following dosing strategy: (**A**) 50 + 3 × 50 g/L and (**B**) 75 + 3 × 75 g/L of the substrate.

Yeast prefers the production of erythritol in an environment of increased osmotic pressure to balance the osmotic pressure between inside and outside of the plasma membrane more effectively, and mannitol is usually only a side product of this biosynthesis. In the present study, the dominant product in the culture broth of both fed-batch processes was mannitol, although significant amounts of erythritol and arabitol were also obtained (Figure 5).

**Figure 6:**
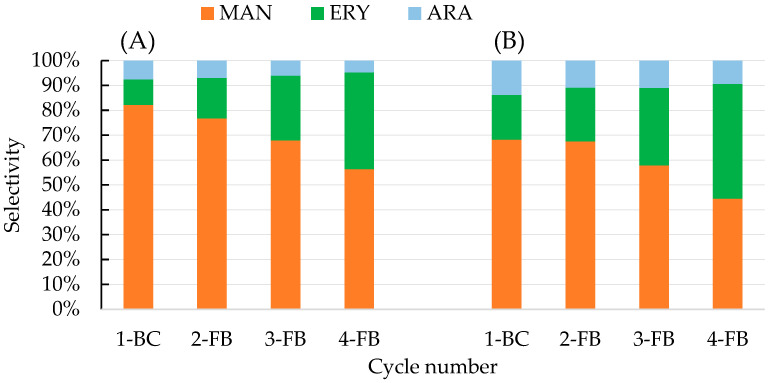
Mannitol—MAN (■), erythritol—ERY (■), and arabitol—ARA (■) production selectivity during fed-batch fermentation in bioreactor cultures of *Y. lipolytica* S3 in production medium with technical glycerol, with the following dosing strategy: (**A**) 50 + 3 × 50 g/L and (**B**) 75 + 3 × 75 g/L of the substrate.

It was observed that the selectivity of mannitol biosynthesis was dependent on glycerol concentration and the cycle of the process (Figure 6). In the process with a lower initial concentration of glycerol (50 g/L), mannitol selectivity reached 82.2% after the first cycle of the process (1-BC), whereas it was lower (68.2%) in the process that started with 75 g/L of glycerol. Next, in both fed-batch processes, mannitol concentration increased, but the selectivity of the cycles (2-4FB) gradually decreased to 56.3% and 44.5%, respectively.

In the total process with 300 g/L of glycerol, 176.4 g/L of polyols was obtained, and the final concentrations of mannitol and erythritol were similar, 78.5 and 81.4 g/L, respectively (Table 3). Application of lower total substrate concentration resulted in lower polyol biosynthesis (140 g/L), but the mannitol production (75.9 g/L) was characterized by a more favorable selectivity index (54.2% for overall process). For comparison, in a fed-batch process performed with the UV-mutant strain *Y. lipolytica* A UV’1 in a medium containing 250 g/L of pure glycerol without addition of NaCl, yeast produced 38.1 g/L of mannitol [29]. Genetically engineered *Y. lipolytica* AIB pAD-UTGut1 was applied in the two-stage process where molasses was used for biomass growth, and next, 150 g/L of raw glycerol was fed, which resulted in production of 100.6 g/L of polyols (including only about 11 g/L of mannitol) [46]. In other studies, addition of Span 20 to the fed-batch culture of an acetate-negative mutant *Y. lipolytica* Wratislavia K1 conducted in glycerol medium (300 g/L) enabled erythritol production to be increased to up to 116.7 g/L, whereas mannitol concentration was 15.4 g/L [26]. In the research aimed at efficient production of erythritol from raw glycerol in fed-batch cultures with different strains of *Y. lipolytica*, 93.5–170 g/L of erythritol and 12.0–40.5 g/L of mannitol were produced from 300 g/L of the substrate [47]. Concluding this stage of research, it should be emphasized that the results of mannitol production obtained in the fed-batch cultures proposed here are definitely higher in comparison to the results obtained by other authors in bioreactor processes involving *Y. lipolytica* yeast (Table 3).

## 3. Materials and Methods

### 3.1. Microorganisms

Twenty-one wild-type yeast representatives of the species *Yarrowia lipolytica*, from the Department of Biotechnology and Food Microbiology, Wrocław University of Environmental and Life Sciences (Poland), were used in this study. Yeasts were isolated from soil (S1→S8, S19) and blue-veined cheese (S9→S18, S20, S21) and identified using API ID 32C strips (bioMérieux SA, Marcy-l’Étoile, France), and data were analyzed with APILAB Plus 3.2.2 software (bioMérieux SA, Marcy-l’Étoile, France) [32]. The strains were maintained on YM-agar slopes, at 4 °C.

### 3.2. Substrates

Technical glycerol (98% wt/wt; POCH, Gliwice, Poland) or raw glycerol from methyl ester production (Wratislawia-Bio, Wrocław, Poland) containing 87.3% wt/wt of glycerol and 4.3% wt/wt of NaCl were used as carbon and energy sources in the media.

### 3.3. Media

The growth medium for inoculum preparation consisted of technical glycerol, 50 g; yeast extract, 3 g; malt extract, 3 g; bacto-peptone, 5 g; and distilled water up to 1 L. The selection of strains for mannitol production was performed in the following screening medium: technical glycerol, 100 g; NH_4_Cl, 2.0 g; KH_2_PO_4_, 0.2 g; MgSO_4_·7H_2_O, 1.0 g; yeast extract, 1.0 g; and CaCO_3_, 2.5 g in 1 L of tap water (see Figure 1). The effect of NaCl on growth and mannitol production was tested in screening medium with 150 g of technical glycerol and addition of NaCl, from 0 to 20% (see Figure 3). Batch mode was conducted in a production medium that contained technical or raw glycerol, 50–250 g/L; NH_4_Cl, 2.0; KH_2_PO_4_, 0.2; MgSO_4_·7H_2_O, 1; and yeast extract, 1 g in 1 L of tap water (see Figure 2). Fed-batch culture was performed in production medium with total technical glycerol concentration of 200 or 300 g/L obtained by adding glycerol in portions, 50 or 75 g/L every 48 h (see Figure 4).

### 3.4. Culture Conditions

The selection of strains and determination of the effect of NaCl on mannitol production were performed in triplicate in flasks containing 50 mL of screening medium with initial pH = 3.0, for 7 days, at 30 °C on a rotary shaker (New Brunswick-Scientific Co.; Edison, NJ, USA) at 160 rpm. The inoculum cultivations were conducted for 72 h in 0.3 L flasks containing 0.1 L of a growth medium on a rotary shaker as described above.

Batch and fed-batch cultures were performed in appropriate production medium in a 5 L BIOSTAT B-PLUS bioreactor (Sartorius Stedim Biotech GmbH, Goettingen, Germany) with a working volume of 2.0 L at 30 °C. The aeration rate was 0.8 vvm, and the stirrer speed was adjusted to 800 rpm. The pH 3.0 was automatically maintained by addition of 20% NaOH. Production medium was inoculated with 10% of the inoculum culture. Samples from the bioreactor were collected 1–2 times per day. Cultures were carried out until complete substrate depletion was achieved in the production medium.

### 3.5. Effect of Osmotic Pressure

The osmotic pressure was measured in the screening medium with NaCl (0 to 20%) and production medium containing 50–250 g/L of technical glycerol, based on the freezing point method using an osmometer (Marcel OS 3000, Warszawa, Poland) and expressed as Osm/kg (osmol/kg).

### 3.6. Analytical Methods

Yeast concentration was determined by dry cell weight analysis. A 10 mL volume of cell suspension from the culture was washed twice with water (2 × 2.5 mL). After centrifuging (at 5000 rpm for 5 min), the biomass was separated using a 0.45 μm pore-sized membrane filter (Merck Millipore, Burlington, Massachusetts, USA) dried at 105 °C until a constant weight was reached, cooled in a desiccator, and weighed.

Mannitol, erythritol, glycerol, arabitol, and citric acid concentrations were determined using the HPLC method (Dionex—UltiMate 3000 LC Systems, Thermo Fisher Scientific, Waltham, MA, USA) on a HyperRez XP Carbohydrate H+ column (Thermo Fisher Scientific, Waltham, MA, USA) coupled to a UV detector (λ = 210 nm) and an RI detector (Shodex, Resonac Europe GmbH, Munich, Germany). The column was eluted at 65 °C with 20 mM trifluoroacetic acid solution at a flow rate of 0.6 mL/min.

### 3.7. Statistical Analysis

Results of the study were analyzed statistically using Statistica 10.0 software (StatSoft, Tulsa, OK, USA).

Cluster analysis (Ward’s method) was used in order to find natural groups among the isolates when mannitol level and erythritol production were taken into account. Squared Euclidean distance was considered as a measure of proximity between strains. One-way analysis of variance was performed to detect significant differences in the data (mannitol or erythritol production) depending on the yeast strain used in flask and batch cultures. Homogeneous groups were determined using Duncan’s test (*p* ≤ 0.05).

## 4. Conclusions

Polyols can replace traditional and controversial sweeteners, and the growing nutritional awareness of consumers as well as the rapidly increasing number of people suffering from diabetes create an increasing demand for such substances. The production of polyols by *Y. lipolytica* yeast is one of the possible solutions for this problem. For over a decade, intensive research has been carried out on the biosynthesis of erythritol by *Y. lipolytica* yeast, but there are still not many studies on the production of mannitol by yeasts of this species.

Out of the 21 *Y. lipolytica* strains tested in this study, the S3 strain was identified as the best producer of polyols such as mannitol and erythritol. It is worth noting that the S3 strain selected in this study was able to produce mannitol as a predominant product, which represented up to 70% of the total amount of polyols. It was also demonstrated that the proposed breeding system allowed for an almost two-fold increase in mannitol production—from 44.6 g/L in the batch culture with 200 g/L of glycerol to 75.9 g/L in the fed-batch process with 200 g/L of glycerol. In addition, the selected S3 strain was characterized by significant resistance to high salt concentrations in the environment and, therefore, to high osmotic pressure. This finding might be of high interest, as it enables efficient production of mannitol using raw glycerol, which usually contains some amounts of salt.

## Figures and Tables

**Table 1:** Comparison of polyol production by selected strains of *Y. lipolytica* in bioreactor batch cultures with technical and raw glycerol.

Strain	MAN * (g/L)	ERY * (g/L)	ΣPol (g/L)	MAN/ΣPol (%)	Y _ΣPol/S_ (g/g)
Technical glycerol					
S2	39.9 ^b^	45.2 ^a^	85.1	46.8	0.57
S3	47.9 ^a^	41.7 ^b^	89.6	53.5	0.60
S4	46.4 ^a^	36.5 ^c^	82.9	56.0	0.55
Raw glycerol					
S2	22.7 ^b^	52.6 ^a^	75.3	30.1	0.50
S3	26.3 ^a^	35.5 ^c^	61.8	42.6	0.41
S4	22.4 ^b^	47.8 ^b^	70.2	32.0	0.47

ΣPol—total polyols (g/L); Y _ΣPol/S_—yield of mannitol in terms of total substrates produced (g/g); * Mean values in the same column with different letters (^a,b,c^) differ significantly at *p* ≤ 0.05. Culture condition: glycerol concentration of 150 g/L.

**Table 2:** Production of mannitol (MAN), erythritol (ERY), arabitol (ARA), citric acid (CA), and biomass (X) by *Y. lipolytica* S3 depending on the initial technical glycerol concentration in bioreactor batch cultures.

Technical Glycerol	Water Activity	Time	X *	MAN *	ERY *	ARA *	CA *	ΣPol	MAN /ΣPol	Y _ΣPol/S_
(g/L)	(OsMol/kg)	(h)	(g/L)					(g/L)	(%)	(g/g)
50	0.77	45	23.3 ^c^	15.7 ^f^	1.5 ^f^	2.5 ^f^	0.5 ^f^	19.7	79.7	0.39
75	1.03	48	20.3 ^d^	19.3 ^e^	5.1 ^e^	3.9 ^e^	2.3 ^e^	28.3	68.2	0.38
100	1.35	76	21.3 ^d^	32.1 ^d^	22.6 ^d^	5.5 ^d^	2.3 ^e^	60.2	53.3	0.60
125	1.93	72	25.6 ^ab^	41.8 ^c^	23.4 ^d^	8.3 ^c^	4.4 ^c^	73.5	56.0	0.59
150	2.24	75	28.0 ^a^	47.4 ^b^	41.2 ^c^	10.0 ^b^	7.3 ^a^	98.6	48.1	0.66
175	2.79	100	25.0 ^b^	54.7 ^a^	52.9 ^b^	10.1 ^b^	6.6 ^b^	117.2	46.7	0.67
200	3.01	98	24.5 ^b^	44.6 ^bc^	87.3 ^a^	10.8 ^a^	3.2 ^d^	142.7	31.3	0.71
250	3.53	100	0.0 ^f^	0.0 ^g^	0.0 ^f^	0.0 ^g^	0.0 ^g^	-	-	-

ΣPol—total polyols (g/L); Y _ΣPol/S_—yield of mannitol in terms of total substrates produced (g/g); * Mean values in the same column with different letters (^a–g^) differ significantly at *p* ≤ 0.05.

**Table 3:** Comparison of erythritol (ERY), mannitol (MAN), arabitol (ARA), and total polyol (ΣPol) produced by various *Yarrowia lipolytica* yeast strains during growth in a medium with a large amount of pure/technical or raw glycerol of fed-batch fermentations.

*Yarrowia lipolytica* Yeast Strain	Substrate: Glycerol (g/L)	Time (h)	MAN (g/L)	ERY (g/L)	ARA (g/L)	ΣPol (g/L)	MAN/ΣPol (%)	Y _ΣPol/S_ (g/g)	References
A UV’1	Pure (250)	133	38.1	91.6	-	129.7	0.29	0.52	[29]
A 15	Pure (250)	150	41.4	67.5	-	108.9	0.38	0.44	
Wratislavia K1	Pure (300)	100	15.4	116.7	-	132.1	11.7	0.44	[26]
AIB	Crude (150)+ 60 g/L molasses		12.6	56.7	6.0	74.2	20.8	0.49	[46]
AIB pAD-UTGut			11.0	82.2	7.5	100.7	10.9	0.67	
CICC 1675	Pure (275)	220	41.2	92.5	-	133.7	30.8	0.49	[28]
1.22	Raw (300)	168	34	93.5	-	127.5	26.7	0.43	[47]
A-101	Raw (300)	168	27	137.0	-	164.0	16.5	0.55	
Wratislavia 1.31	Raw (300)	168	23	132.0	-	155.0	14.8	0.52	
Wratislavia AWG7	Raw (300)	168	19	124.0	-	143.0	13.3	0.48	
Wratislavia K1	Raw (300)	168	12	170.0	-	182.0	6.6	0.61	
8661 UV1	Raw (300)	168	40.5	113.0	-	153.5	26.4	0.51	
S3	Technical (200)	240	75.9	52.4	11.7	140.0	54.2	0.70	present study
	Technical (300)	220	78.5	81.4	16.5	176.4	44.5	0.59	

## Data Availability

Not applicable.

## Sample Availability

Not available.
